# The probability of cerebral amyloid angiopathy according to the Simplified Edinburgh CT criteria in a large, unselected lobar intracerebral hemorrhage population

**DOI:** 10.1007/s00234-025-03555-8

**Published:** 2025-02-12

**Authors:** Amir Hillal, Trine Apostolaki-Hansson, Birgitta Ramgren, Björn Hansen, Bo Norrving, Johan Wassélius, Teresa Ullberg

**Affiliations:** 1 Department of Medical Imaging and Physiology, Skåne University Hospital, Lund, 221 85 Sweden; 2 Department of Neurology, Skåne University Hospital, Malmö, Sweden; 3 Department of Clinical Sciences Lund, Lund University, Lund, Sweden

**Keywords:** Intracerebral hemorrhage, Non-contrast Computed Tomography, Cerebral amyloid angiopathy, Hematoma volume

## Abstract

**Purpose:**

Early identification of the underlying cause of intracerebral hemorrhage (ICH) is important for treatment and prognosis. This study aims to investigate the association of hematoma volume and other clinical parameters on the distribution of cerebral amyloid angiopathy (CAA) probability according to the simplified Edinburgh CT criteria in a large, unselected intracerebral hemorrhage (ICH) population.

**Method:**

Patients with spontaneous ICH residing in Skane county registered with clinical data in the Swedish Stroke Register 2016–2020 were included. Radiological parameters were evaluated using baseline non-contrast CT (NCCT) for categorization according to the simplified Edinburgh CT criteria by the presence of subarachnoid hemorrhage (SAH) and fingerlike-projections (FLP). Multivariable logistic regression analysis was used to determine factors associated with an increased (intermediate/high) CAA probability.

**Results:**

Of 666 patients with lobar ICH, 190 (29%) had *high*, 92 (14%) had *intermediate*, and 384 (58%) had *low* CAA probability. Patients with increased CAA probability presented more often with decreased level of consciousness, larger hematoma volumes, and higher 90-day mortality. Baseline hematoma volume [10-30 ml (OR = 4.03;95%CI: 2.26–7.19); 30-80 ml (OR = 12.00;95%CI:7.26–22.53); >80 ml (OR = 30.00;95%CI:15.94–59.09)], female sex (OR = 1.58;95%CI:1.08–2.32) and age (OR = 1.04;95%CI:1.02–1.06) were associated with an increased odds of having an increased CAA probability.

**Conclusion:**

We identified a strong association between baseline hematoma volume and an increased probability of CAA in lobar ICH patients on NCCT, indicating that large hematoma volumes alone may contribute to the occurrence of FLP and SAH, and act as a confounder for the simplified Edinburgh CT criteria. Validation against MRI is warranted.

## Introduction

Intracerebral hemorrhage (ICH) accounts for up to 30% of all strokes worldwide and has a 30-day fatality rate of up to 40% [1, 2]. Early identification of ICH etiology is important for prognostication and treatment to prevent hematoma expansion, the most important modifiable predictor of patient outcome [3, 4].

Cerebral amyloid angiopathy (CAA) is classically associated with lobar ICH and accounts for up to 20% of all spontaneous ICHs [5]. Early identification of CAA is important since it constitutes a significant risk of hemorrhage recurrence, and the time window to prevent hematoma expansion may be longer [4]. Introduced in 1995 and later modified in 2010, the Boston criteria is the gold standard for an image-based diagnosis of CAA based on the detection of cerebral microbleeds and cortical superficial siderosis on magnetic resonance imaging (MRI) [6–10]. In 2022, the Boston criteria 2.0 was introduced, incorporating clinical, imaging, and pathological parameters, based on the original and modified criteria aiming to refine and advance the diagnostic approach to CAA [10]. The Boston 2.0 criteria includes leptomeningeal and white matter characteristics for the diagnosis of probable and possible CAA. However, MRI is less often used for ICH diagnosis in routine healthcare, whereas non-contrast computed tomography (NCCT) is more widely accessible. In 2018, Rodrigues et al. proposed the Edinburgh criteria to identify lobar ICH associated with CAA by combining computed tomography (CT) features with information on the apolipoprotein E (APOE) genotype [11]. The study identified that the co-occurrence of subarachnoid hemorrhage (SAH) with either the APOE ε4 allele or “finger-like projections” (FLP) demonstrated nearly 100% sensitivity for CAA. However, a major limitation in its clinical application is the frequent unavailability of APOE-status at the time of CT examination. The simplified Edinburgh CT criteria was later described comprising of only NCCT features without necessitating APOE-status (Table 1), an approach that proved effective in accurately identifying probable CAA cases in patients with lobar ICH, similar to the performance of the Boston criteria [12, 13].

**Table 1:** Lobar intracerebral hemorrhage imaging features for the final study population

Imaging features of lobar ICH (*n* = 666)	Frequency (percentage)
Multifocal	84 (12.6%)
Finger-like projections	190 (28.5%)
Subarachnoid component	234 (32.6%)
Intraventricular extension	217 (32.6%)
Hydrocephalus	180 (27.1%)
Abbreviations: ICH = Intracerebral hemorrhage	

Sembil JA et al. demonstrated good discrimination between the simplified Edinburgh CT criteria and the MRI Boston criteria in validating the probability of CAA (AUC 0.74, 95% CI 0.67–0.81) [13]. In comparison to the Boston criteria, the simplified Edinburgh CT criteria showed a specificity of 87.1% and a sensitivity of 80.9% for the diagnosis of probable CAA [13]. In the decision analysis conducted to assess the overall utility of the simplified Edinburgh criteria, it was observed that FLP and SAH demonstrated a clinical net benefit when used to rule in probable CAA and avoid false positives. These findings suggest that incorporating FLP and SAH into the diagnostic process can enhance clinical utility by minimizing the occurrence of false positives. This may assist physicians with decision-making regarding additional imaging and prognostication. However, no clinical net benefit was identified for ruling out CAA when compared to a default strategy of assuming that either no or all patients have probable CAA, suggesting that the simplified Edinburgh criteria may not provide a clear advantage in terms of clinical utility.

Furthermore, a study by Shwarz G. et al. indicated that the CT biomarkers, FLP and SAH, included in the simplified Edinburgh CT criteria could help rule in probable CAA in ICH patients, with a positive likelihood ratio of + 2.3 (95% CI: 1.2–4.6). However, the absence of these CT biomarkers was not helpful in ruling out probable CAA, as evidenced by a negative likelihood ratio of 0.8 (95% CI: 0.7–1.0) [14]. Additionally, a recent study by Grangeon L et al. showed that the addition of a new CT biomarker (ICH cortical involvement) to the previously known CT biomarkers (SAH, FLP) in the simplified Edinburgh CT criteria resulted in an increased AUC from 0.760 to 0.808 ( 95%CI: 0.714–0.901) for the diagnosis of CAA, MRI was used for the final assessment in this study [15].

The aim of this study was to investigate the association of hematoma volume and other clinical parameters on the distribution of CAA probability according to the simplified Edinburgh CT criteria in a large, unselected lobar ICH population.

## Methods

### Study design and database

This was an observational cohort study based on data from the Swedish Stroke Register, Riksstroke. Data from Riksstroke were collected from 8 primary stroke centers and 1 comprehensive stroke center in the Skåne region. Riksstroke is the Swedish quality register for stroke care with a coverage of > 90% of hospitalized stroke patients in Sweden [16]. Patients registered in Riksstroke are informed of their registration and of the future handling of their patient data for research purposes. All-cause mortality was obtained from the Swedish Cause of Death Register, with a coverage of > 98% [17].

### Clinical data

Clinical data for patients included in this study were collected from Riksstroke, with the following variables on patient and clinical characteristics: age, sex, diabetes mellitus, previous stroke, presence of antithrombotic medication prior to ICH, level of consciousness at hospital admission (alert/drowsy/comatose), use of neurosurgery, and admission to an intensive care unit (ICU).

### Image data and evaluation

CT images were acquired from the regional Picture Archiving and Communication System (PACS), which contains radiological images and data from all 9 hospitals in the Skane region. Scanners from all major manufacturers were used to perform NCCT images. Thin axial reconstructions with a slice thickness of 0.5–1 mm were used for the analyses. Hematoma volumes were measured by manual segmentation using the Sectra Volume Measurement tool (Sectra IDS7, Sectra, Linköping, Sweden). Volume estimation was also performed using the ABC/2 method [18, 19]. The NCCT scans for all patients with lobar ICH were examined and evaluated by a radiology resident with one year of neuroradiology experience. The initial 500 CT scans, of which 190 patients had lobar ICH, were also independently evaluated by a senior neuroradiologist with more than 20 years of experience for inter-rater agreement assessment. In case of disagreement between the readers, a consensus decision was made.

**Fig. 1:**
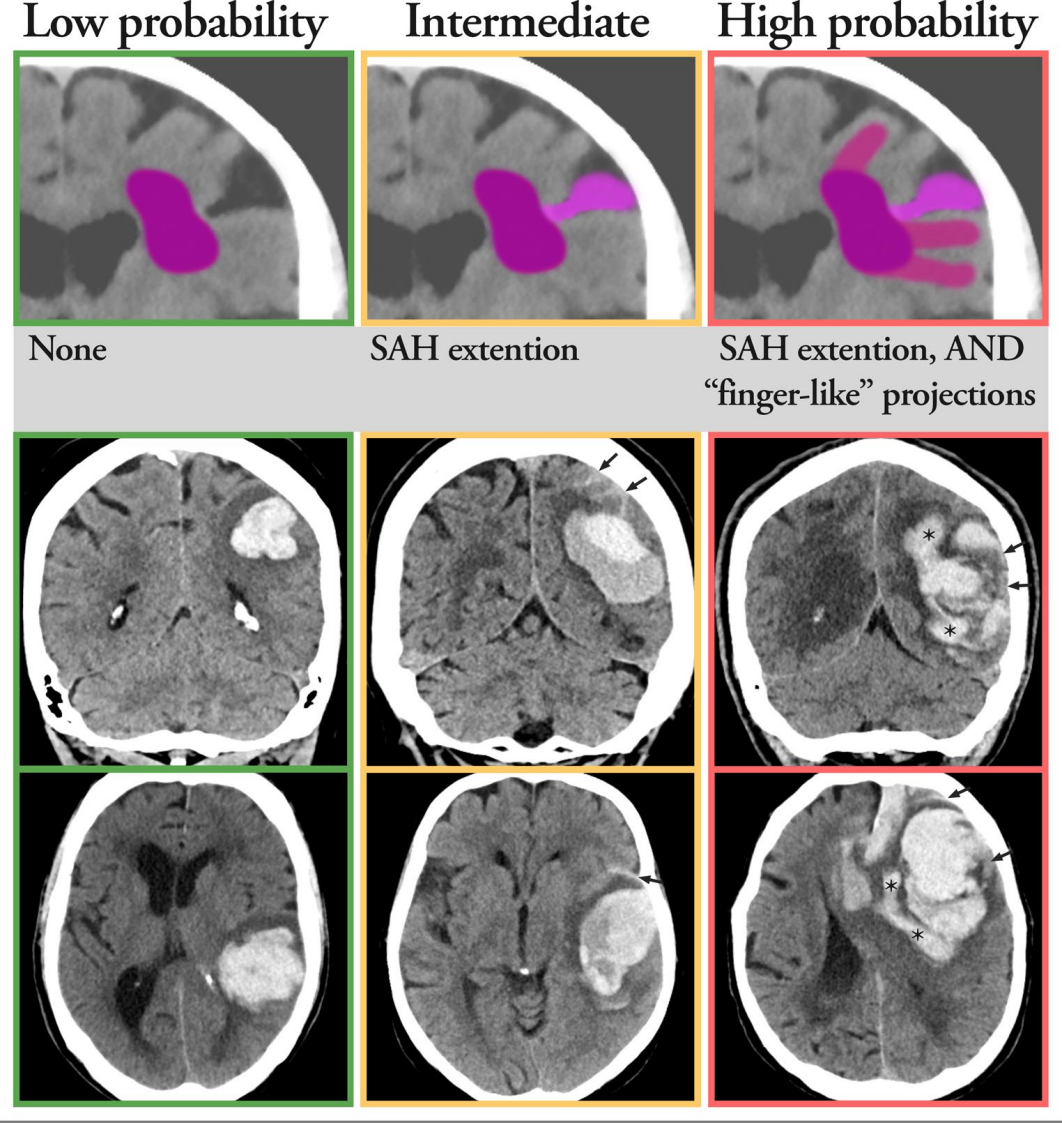
Illustrations of the imaging findings used for the simplified Edinburgh CT criteria and typical imaging examples for low, intermediate, and high probability of cerebral amyloid angiopathy (CAA). Top row illustrating the imaging features, and middle and bottom rows illustrating the typical clinical imaging examples in coronal and transaxial projections for low, intermediate, and high probability of CAA. Abbreviations: CT = Computed tomography, SAH = Subarachnoid hemorrhage

Image evaluation included the following parameters: ICH location (lobar and/or deep or only intraventricular hemorrhage (IVH)), lateralization, single or multifocal (defined as multiple ICHs without any connection), presence of SAH (yes/no), presence of known or newly diagnosed vascular malformation at the time of stroke onset (yes/no), presence of FLP (yes/no) (determined by the appearance of elongated extensions arising from the lobar hematoma that are longer than their width) [11], presence of subdural component (yes/no), presence of intraventricular extension (yes/no), midline shift (measured in mm), or hydrocephalus (yes/no). Image evaluation included as well determining CAA probability in lobar ICHs according to simplified Edinburgh CT criteria [13], illustrated in Fig. 1: Low CAA probability (Lobar ICH, without associated SAH), Intermediate CAA probability (Lobar ICH with associated SAH), High CAA probability (Lobar ICH with associated SAH and presence of FLP).

In cases where follow-up imaging was available, a follow-up NCCT performed within one week of the initial NCCT examination was analyzed using the same parameters.

### Study population

All patients registered in Riksstroke within the Skåne region between 2016 and 2020 with spontaneous non-traumatic lobar intracerebral hemorrhage were included in this study based on the following inclusion criteria:

- Age ≥ 18 years.
- Availability of the baseline NCCT image in the regional PACS.
- Spontaneous, supratentorial, lobar ICH (non-traumatic and not caused by an underlying brain tumor or other known structural lesion).

### Exclusion criteria were the following

- Major technical limitations on the baseline CT (such as severe motion artifacts, or lack of preoperative images).
- Lack of clinical data.

### Statistical analysis

IBM SPSS version 27 was used for all statistical analyses. Cohen’s kappa was used to assess inter-rater agreement for the presence of SAH and FLP on NCCT images. Lobar ICHs were categorized into a low, intermediate, or high probability of having characteristics associated with a probable CAA diagnosis in accordance with the simplified Edinburgh CT criteria [11]. Binary logistic regression was used to investigate the odds ratios for the diagnosis of if the following variables were associated with an intermediate or high CAA probability at baseline CT: age, gender, previous stroke, known OAC treatment, and hematoma volume. Low CAA probability was set as the reference variable. Independent samples median test was used to estimate differences in the median hematoma volume between patients with- and without OAC prior to lobar ICH. Pearson chi-square test was used to determine the differences in mortality between patients with low, intermediate, and high probability of CAA.

### Ethical considerations

Approval of this study was received by the Swedish Ethical Review Authority (#2020–06800).

## Results

**Fig. 2:**
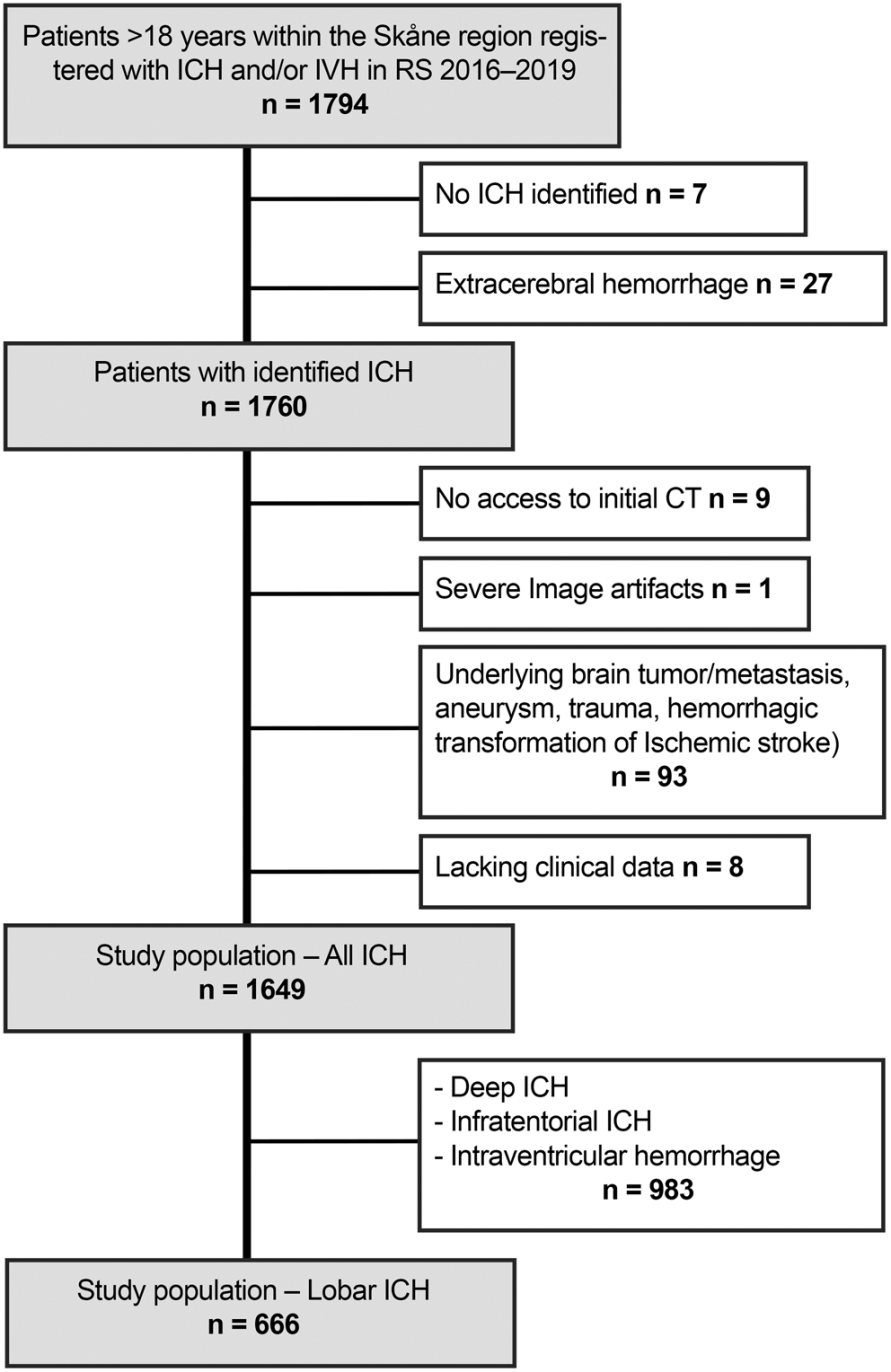
Consort diagram illustrating the exclusion criteria accounting for the total number of patients with spontaneous non-traumatic intracerebral hemorrhage (ICH) in the Skane region between 2016–2020 (*n* = 1649 ICH: 666 lobar ICH). Abbreviations: ICH = Intracerebral hemorrhage, IVH = Intraventricular hemorrhage, CT = Computed tomography, RS = Riksstroke

There were 1649 patients with spontaneous non-traumatic intracerebral hemorrhage within the Skåne region registered in Riksstroke between 2016 and 2020 (Fig. 2). The final study population consisted of 666 patients with lobar ICH; the remaining 983 patients had infratentorial ICH (*n* = 204), IVH only (*n* = 48), and deep ICH (*n* = 731). The NCCT imaging features of all lobar ICH patients are shown in Table 1. The inter-rater analysis of the 190 lobar ICHs that were screened by both radiologists showed an excellent agreement (Kappa 0.88) for supratentorial ICH location (lobar/deep), and a moderate agreement for evaluating the presence of SAH (Kappa 0.55) and for the presence of FLP (Kappa 0.49).

### Evaluation of CAA probability

Out of the 666 patients with lobar ICH, 190 patients (28.5%) had associated SAH and FLP on baseline NCCT and were consequently categorized as having a high probability of CAA according to the simplified Edinburgh CT criteria (Table 2). An intermediate probability of CAA was identified in 92 patients (13.8%) who had associated SAH without FLP on baseline NCCT. The remaining 384 patients (57.7%) had no SAH on baseline NCCT and were therefore classified as having a low probability of CAA.

**Table 2:** Categorization of initial and follow-up lobar intracerebral hemorrhage CT scans according to the simplified Edinburgh CT criteria

Simplified Edinburgh CT Criteria			
CAA grade	Radiological definition	Distribution at Baseline CT (*n* = 666)	Distribution at Follow-up CT (*n* = 208)
Low CAA probability	Lobar ICH without SAH	384(57.7%)	125 (60.1%)
Intermediate CAA probability	Lobar ICH with: • SAH	92(13.8%)	26 (12.5%)
High CAA probability	Lobar ICH with: • SAH, and • Fingerlike projections	190 (28.5%)	57 (27.4%)

Abbreviations: CT = Computed tomography, CAA = Cerebral amyloid angiopathy, ICH = Intracerebral hemorrhage, SAH = Subarachnoid hemorrhage

**Fig. 3:**
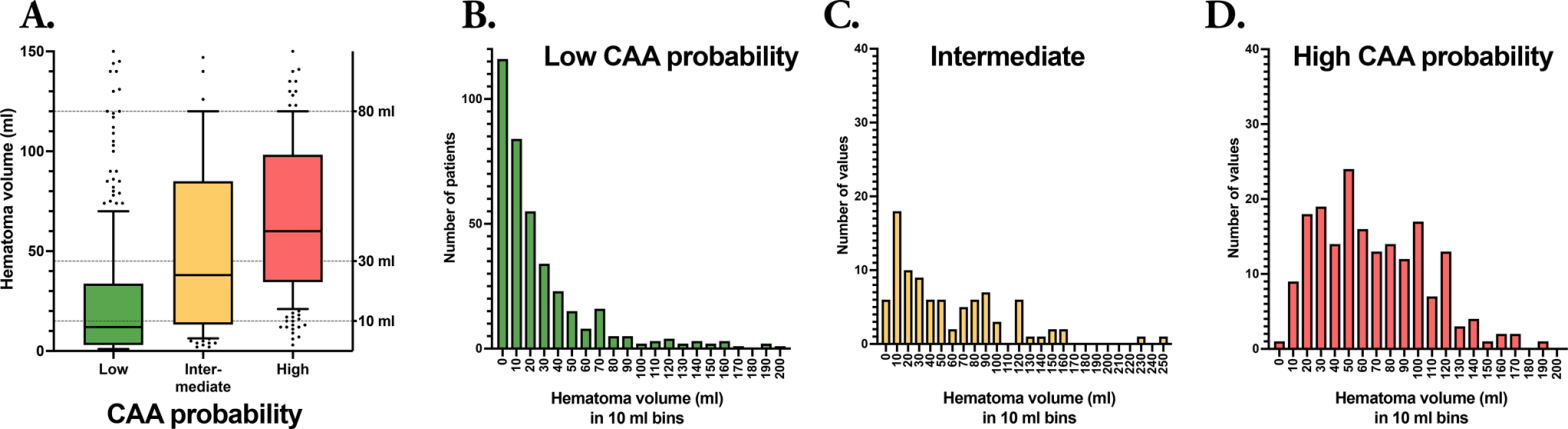
Panel **A**: Box plot showing the distribution and variation of baseline hematoma volume for intracerebral hemorrhage (ICH) patients with a low, intermediate, and high probability of cerebral amyloid angiopathy (CAA). Boxes contain 50% of each group, with median indicated as the line within each box and whiskers indicate the 10th and 90th percentiles. Panels **B**-**D** illustrates the distribution of lobar ICH cases with low (**B**), intermediate (**C**) and high (**D**) CAA probability according to simplified Edinburgh CT criteria in 10 ml bins (note that the range of the y-axis is different in panel **B** compared to **C** and **D**). Abbreviations = CAA = Cerebral amyloid angiopathy

**Fig. 4:**
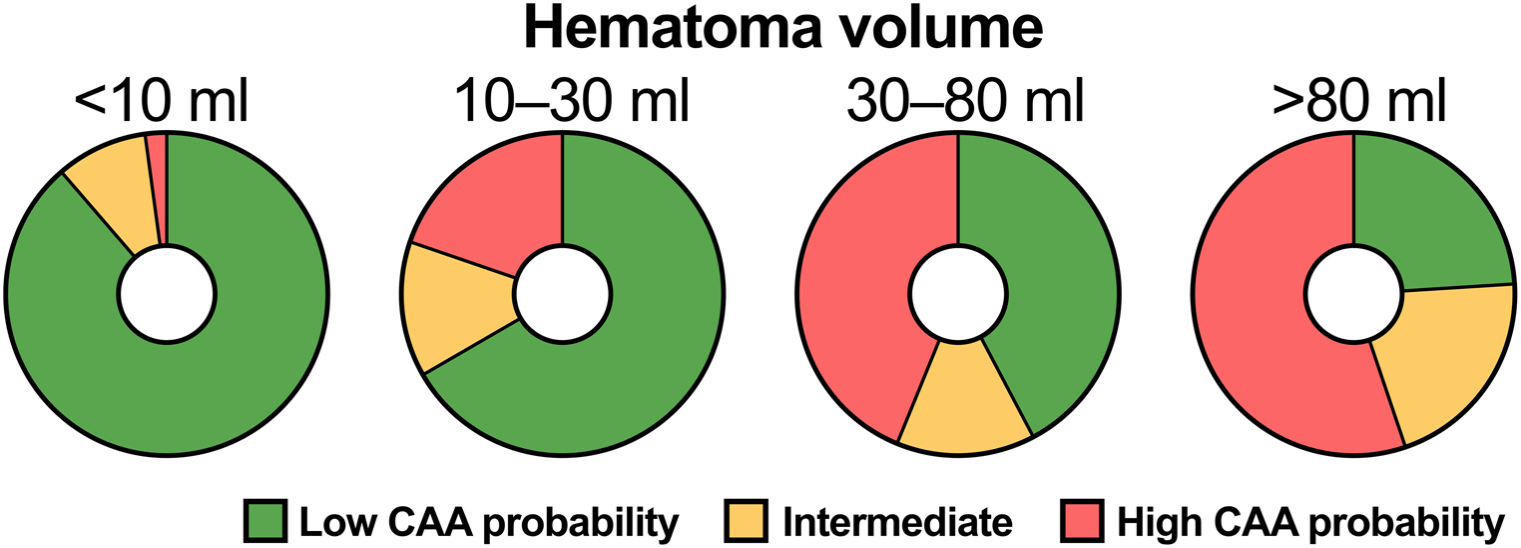
The proportion of cerebral amyloid angiopathy probability according to the simplified Edinburgh CT criteria in patients with lobar intracerebral hemorrhage showing volumes within the four predefined ranges < 10 ml, 10–30 ml, 30–80 ml, and > 80 ml. Abbreviations: CAA = Cerebral amyloid angiopathy

Patients with an intermediate and high probability of CAA were older, more often female, and more often had a history of diabetes and previous stroke compared to patients with a low probability of CAA (Table 3). Patients with an intermediate and high probability of CAA presented more frequently with an altered level of consciousness at admission, larger ICH volumes (Figs. 3 and 4), intraventricular hematoma extension, hydrocephalus, and were less likely to have received neurosurgical interventions and/or intensive care. Ongoing OAC treatment was most frequent in patients with a low probability of CAA (28.9%), compared to those with an intermediate (17.4%) and high (15.8%) probability of CAA. The proportion of patients who died within 90 days was higher in patients with an intermediate (43.5%) and high (52.1%) probability of CAA compared to patients with a low probability of CAA (18.8%) (*p* < 0.001).

**Table 3:** Clinical parameters for patients with lobar intracerebral hemorrhage with a low, intermediate, and high probability of cerebral amyloid angiopathy according to the simplified Edinburgh CT criteria

Clinical Parameters	Low CAA probability (*n* = 384)	Intermediate CAA probability (*n* = 92)	High CAA probability (*n* = 190)
Sex	M: 230 (60%) F: 154 (40%)	M: 49 (53%) F: 43 (47%)	M: 81 (43%) F: 109 (57%)
Age, median (IQR)	74 (17)	77 (12)	80 (13)
Diabetes mellitus	57 (15%)	14 (15%)	31 (16%)
Previous stroke	90 (24%)	24 (26%)	50 (26%)
OAC at onset	111 (29%)	16 (17%)	30 (16%)
Level of consciousness at first assessment	Alert: 267 (70%) Drowsy: 74 (19%) Comatose: 40 (11%)	Alert: 48 (52%) Drowsy: 21 (23%) Comatose: 21 (23%)	Alert: 72 (38%) Drowsy 60 (32%) Comatose: 55 (30%)
MRI brain performed during time of care	89 (23%)	14 (15%)	19 (10%)
Neurosurgical intervention	25 (7%)	7 (7.7%)	9 (5%)
ICU treatment	28 (7%)	6 (7%)	10 (5%)
ICH volume (median)	12 mL	38 mL	60 mL
IVH extension	76 (20%)	42 (46%)	99 (52%)
Hydrocephalus	54 (14%)	38 (41%)	88 (46%)
Death within 90 days	72 (19%)	40 (44%)	99 (52%)

Abbreviations: CAA = Cerebral amyloid angiopathy, F = Female, ICH = Intracerebral hemorrhage, ICU = Intensive care unit, IQR = Interquartile range, IVH = Intraventricular hemorrhage, M = male, MRI = Magnetic resonance imaging, OAC = Oral anticoagulation

Out of the 666 patients with lobar ICH, 122 patients (18.3%) had performed a brain MRI during time of care. In addition, only 41 patients (6.2%) received any neurosurgical intervention, with a successively lower proportion of patients receiving neurosurgical care as the probability of CAA increased.

A multivariable logistic regression analysis was used to determine imaging aspects and clinical factors associated with an increased odds of an intermediate or high probability of CAA (Table 4). After adjusting for covariates in the regression analysis, baseline hematoma volumes of 10–30 ml (OR = 4.03 [95% CI: 2.26–7.19]), 30–80 ml (OR = 12.79 [95% CI: 7.26–22.53]), and > 80 ml (OR = 30.69 [95% CI: 15.94–59.09]), as well as female sex (OR = 1.58 [95% CI: 1.08–2.32]) and age (OR = 1.04 per year [95% CI: 1.02–1.05]), were associated with higher odds of having an increased CAA probability. Ongoing OAC treatment prior to ICH was associated with lower odds of having an increased probability of CAA (OR = 0.27 [95% CI: 0.17–0.44]). Independent sample median test showed no significant difference in the median volume of baseline hematoma between patients with and without OAC treatment prior to lobar ICH (*p* = 0.65).

**Table 4:** Logistic regression analysis showing the Odds Ratios for patients with an *intermediate* or *high* probability of cerebral amyloid angiopathy (CAA) based on baseline CT. *Low* probability of CAA is set as the reference

Variables	OR	95% CI	*p*-value
Simplified Edinburgh CT criteria: Low CAA probability (reference), to Intermediate- and High CAA probability			
Age	1.040	1.022–1.058	< 0.001
Sex (F)	1.58	1.076–2.32	0.02
Previous stroke	1.23	0.791–1.903	0.36
OAC at onset	0.273	0.170–0.438	< 0.001
Baseline hematoma volume: 10–30 ml 30–80 ml > 80 ml	4.03 12.79 30.69	2.26–7.19 7.26–22.53 15.94–59.09	< 0.001 < 0.001 < 0.001

Abbreviations: CI = Confidence interval, CAA = Cerebral amyloid angiopathy, CT = Computed tomography, F = Female, OAC = Oral anticoagulation, OR = Odds ratio

### Follow-up NCCT

A follow-up NCCT within one week after lobar ICH was available for 31.2% of all patients included in this study (208/666). Among the lobar ICH population, 19.2% of patients had died within one week (128/666), with 53.9% having a high probability of CAA, and only 11.7% of these patients underwent a follow-up NCCT. On follow-up NCCT, 125/208 (60.1%) patients were classified as having a low probability of CAA, 26/208 patients (12.5%) as having an intermediate probability of CAA, and 57/208 patients (27.4%) as having a high probability of CAA (Table 2).

On follow-up NCCT, SAH +/- FLP were newly identified in 18 patients that previously lacked these features, thereby increasing their CAA probability grade (Table 5). These 18 patients more often had ongoing OAC treatment prior to lobar ICH, larger final hematoma volumes (median: 53 ml, compared to 23 ml), a higher rate of hematoma expansion (55.6%, compared to 18%), and a higher proportion of death within 90 days (28%, compared to 15%). Furthermore, on follow-up NCCT, SAH had resolved in 13 patients that previously had this feature.

**Table 5:** Clinical and imaging parameters for patients with lobar intracerebral hemorrhage with an *unchanged* and *upgraded* probability of cerebral amyloid angiopathy at follow-up CT according to the simplified Edinburgh CT criteria

Clinical and imaging Parameters (follow-up CT)	Unchanged CAA probability (*n* = 190)	Increased CAA probability (*n* = 18)
Hematoma volume (median)	23 ml	53 ml
Hematoma expansion	34 (18%)	10 (56%)
OAC treatment at onset	47 (28%)	9 (50%)
Death within 90 days	28 (15%)	5 (28%)

Abbreviations: CAA = Cerebral amyloid angiopathy, CT = Computed tomography

## Discussion

This study shows the distribution of CAA probability according to the simplified Edinburgh CT criteria in a large unselected regional ICH population in Sweden. In this highly representative population, we show that 42% of patients with lobar ICH have an increased probability of CAA based on the simplified Edinburgh CT criteria, of which 14% were classified as having an *intermediate* probability and 29% as having *high* probability. In comparison, results from the initial study on the simplified Edinburgh CT criteria (Sembill et al.) involving a cohort of 210 patients with lobar ICH showed that 35% of patients had *low* CAA probability, 32% were classified as having an *intermediate* probability, and 33% were classified as having a *high* probability according simplified Edinburgh CT criteria [13]. This slight discrepancy in proportions among probability groups may arise from differences in the studied cohorts. Our observational cohort study, being large and unselected, contrasts with the cohort in the study performed by Sembill et al., which comprises a lower number of included patients from a prospective institutional stroke registry at a Neurology department in a University Hospital. However, despite these differences, the inter-rater agreement was similar to ours.

In patients with lobar ICH, hematoma volume was strongly associated with an increased odds of having an intermediate and high CAA probability according to the simplified Edinburgh CT criteria. Baseline hematoma volumes between 30 and 80 ml were associated with a 13-fold increase in CAA probability, and a 30-fold increase in CAA probability was seen for ICH volumes > 80 ml. The strong association between CAA probability and ICH volume raises the possibility that large hematoma volumes alone may contribute to the occurrence of FLP and SAH, and act as a confounder for the simplified Edinburgh CT criteria. Our results regarding CAA probability and its association with hematoma volume are in line with findings from a retrospective study by Ornello et al. on an unselected ICH population (*n* = 494), of which 178 patients had lobar ICH [20]. Similarly, this study showed that patients with imaging features of a high probability of CAA had larger ICH volumes at baseline, higher in-hospital death, and worse modified Rankin scale scores at discharge [20]. Furthermore, in lobar ICH patients with hereditary CAA, van Ethen et al. showed that hematoma volume was significantly larger in patients with intermediate and high CAA probability according to NCCT. However, the sensitivity differed between large and small bleedings, with 81% sensitivity for ICH volumes ≥ 40 ml and less than 50% for ICH volumes ≤ 15 ml, concluding that the simplified Edinburgh CT criteria is a sensitive diagnostic test in patients with CAA-associated lobar ICH, though it should be applied carefully in cases of small ICH volumes [21]. Our results align with this finding, as we observed a strong association between ICH volume and CAA probability (mean ICH volume; low CAA probability: 27 ml, intermediate CAA probability: 55 ml, high CAA probability: 67 ml).

It has been previously shown that CAA is a common cause of lobar ICH in the elderly [5, 22], it has also been proposed that men may have earlier CAA onsets with more frequent occurrences of ICH compared to women [23]. In our study, age and female sex were significantly associated with an increased odds of having an intermediate and high CAA probability in patients with lobar ICH. Furthermore, patients with high CAA probability more often presented with an altered level of consciousness compared to patients categorized as having a low or intermediate probability of CAA.

Ongoing OAC treatment prior to lobar ICH was more frequent in patients that had a low probability of CAA. In patients with a low probability of CAA, the median baseline hematoma volumes were comparable (*p* = 0.08) between patients with prior OAC (11 ml) and those without (17 ml), Conversely, patients with an intermediate or high probability of CAA and ongoing OAC treatment had higher median baseline hematoma volumes (79 ml) and a greater proportion of death within 90-days (*p* < 0.001) compared to those without treatment ongoing OAC treatment (50 ml; *p* < 0.01).

Follow-up NCCT was only performed in 1/3 of patients, and we noticed that in those follow-up images, SAH +/- FLP was diagnosed in 8.6% of patients that previously lacked these features, thereby increasing their CAA probability grade. These patients specifically had larger final hematoma volumes, a higher rate of hematoma expansion, and a higher rate of death within 90 days. To our knowledge, transition from a lower to a higher CAA probability according to the simplified Edinburgh criteria has not been previously described. This novel finding is interesting since it may represent a limitation in the use of the simplified Edinburgh criteria since the CAA probability may change over time and with repeat imaging.

Factors that were more common in patients who transitioned to a higher probability of CAA were ongoing OAC treatment prior to ICH, larger final hematoma volumes, and higher rates of hematoma expansion. Moreover, patients who transitioned had a higher proportion of death within 90 days. These findings provide additional support for our prior presumption that larger hematoma volumes are associated with a higher probability of CAA. However, it is important to note that this observation is derived from a small number of patients who underwent repeated imaging and is not statistically significant. Hence, it seems reasonable to state that follow-up imaging in patients with ICH should be encouraged to further identify patients with a high probability of CAA, and that further studies are required to determine whether the probability of CAA can change based on follow-up imaging. On follow-up NCCT, SAH had resolved in a few patients that previously had this feature (*n* = 13). This finding is presumably attributed to the natural process of SAH redistribution into the subarachnoid space, and any change in CAA probability in this subgroup of patients appears inconsequential.

Our study shows that the rates of follow-up brain MRI, ICU treatment, and neurosurgical intervention were low, and they successively decreased as the probability of CAA increased. This is of notifiable importance as lobar ICH patients reportedly have a higher ICH recurrence rate compared to their deep ICH counterparts [24, 25], and lobar ICH is more often considered for hematoma evacuation given the accessible hemorrhage location [26, 27].

Lastly, our study shows that lobar ICH patients with a high CAA probability had an increased unadjusted death rate within 90 days (52%) compared to patients with low (19%) and intermediate (44%) probability. Future studies are warranted to determine mortality rates in different CAA risk groups and in patients with lobar versus deep ICH, as data regarding mortality related to these specific populations are scarce.

### Limitations

Firstly, the most important limitation of this study was the lack of MRI studies to allow for the correlation between CT imaging characteristics and the gold imaging standard MRI Boston criteria. Additionally, the prevalence of underlying lesions (such as tumors, arteriovenous malformations and cavernomas), may therefore be underestimated.

Secondly, the proportion of follow-up imaging was low, which limits the possibility to analyze any associations between CAA-characteristics and hematoma expansion. The inter-rater agreement between both radiologists was moderate for evaluating the presence of SAH and FLP. Similar moderate agreements were seen in previous studies of the original Edinburgh and simplified Edinburgh criteria [11, 13]. This result could be due to the fact that FLPs specifically in irregularly shaped ICHs are difficult to define, are subjective, and are prone to observer variability. In addition, the mass effect of a large ICH on the surrounding brain parenchyma could resultantly cause difficulties in the assessment of the presence of SAH.

## Conclusion

In this large, unselected ICH cohort, we identified that 42% of patients with lobar ICH were either classified as having an intermediate or high probability of CAA. Furthermore, a strong association between the baseline hematoma volume and an increased probability of a probable CAA diagnosis was identified, revealing up to a 30-fold increase in the odds of an intermediate or high CAA probability for very large hemorrhages. This indicates that large hematoma volumes alone may contribute to the occurrence of FLP and SAH, and act as a confounder for the existing simplified Edinburgh CT criteria. This presumption requires further validation studies against established MRI criteria for CAA. Furthermore, our findings shed light on the importance of identifying patients with a high probability of CAA given the high risk of ICH recurrence and the high death rates observed in these patients.

## Data Availability

An anonymized dataset, supporting the conclusions of this article may be provided upon reasonable request.
